# An ethnobotanical survey of medicinal plants in Babungo, Northwest Region, Cameroon

**DOI:** 10.1186/1746-4269-6-8

**Published:** 2010-02-15

**Authors:** David J Simbo

**Affiliations:** 1 Department of Bioscience Engineering Faculty of Science, University of Antwerp Groenenborgerlaan 171 2020 Antwerp, Belgium

## Abstract

**Background:**

An ethnobotanical survey was undertaken to record information on medicinal plants from traditional medical practitioners in Babungo and to identify the medicinal plants used for treating diseases.

**Methods:**

Traditional Medical Practitioners (TMP's) who were the main informants were interviewed using semi-structured questionnaires and open-ended conversations. Field trips were made to the sites where TMP's harvest plants.

**Results:**

The survey identified and recorded 107 plants species from 54 plant families, 98 genera used for treating diseases in Babungo. The Asteraceae was the most represented plant family while herbs made up 57% of the total medicinal plants used. The leaf was the most commonly used plant part while concoction and decoction were the most common method of traditional drug preparation. Most medicinal plants (72%) are harvested from the wild and 45% of these have other non medicinal uses. Knowledge of the use of plants as medicines remains mostly with the older generation with few youth showing an interest.

**Conclusions:**

A divers number of plants species are used for treating different diseases in Babungo. In addition to their use as medicines, a large number of plants have other non medicinal uses. The youth should be encouraged to learn the traditional medicinal knowledge to preserve it from being lost with the older generation.

## Background

Ethnobotany is the study of how modern and indigenous societies view and use plants [1]. The use of natural products with healing properties is as old as human civilization and for a long time, minerals, animal and plant products were the main sources of drugs [2]. The World Health Organization (WHO) defines traditional medicine as practices, knowledge and belief systems which uses minerals, plants and animal based remedies, spiritual therapies and exercises to prevent, treat and maintain well being [3]. According to the WHO, about 80% of the population of the world depends on traditional medicine, mostly herbal remedies, for their primary health care needs [4]. The African continent have a long history with the use of plants and in some African countries, up to 90% of the population rely on medicinal plants as a source of drugs [5]. A medicinal plant is any plant, which in one or more of its organs contains active ingredients which can be used for therapeutic purposes or contain foundation compounds that can be used for the synthesis of useful drugs [6]. The absence or inaccessibility of modern healthcare services, affordability, cultural acceptance and, under certain circumstances, effectiveness than their modern counterparts has caused a large percentage of the population to rely mostly on plant based traditional medicines for their primary health care needs. These factors and a growing interest in the use of natural products and folk medicine have resulted to an increase in the demand for medicinal plants [7]. This increase in demand puts a threat on natural resources. Knowledge on the use of medicinal plants is enormous but if this is not rapidly researched and recorded, indications are that it will be lost with succeeding generations [5].

An estimated 25% of prescription drugs and 11% of drugs considered essential by the WHO are derived from plants and a large number of synthetic drugs are obtained from precursor compounds originating from plants [2]. Therefore the documentation of the traditional therapeutic know-how could lead to the discovery of new drugs as well as contribute to the conservation, sustainable management and use of plant resources. Ethnobotanical investigations have been reported for parts of Cameroon [8-10] and parts of the adjacent Bamenda highlands [11] but no investigation has ever been carried out in Babungo. It is therefore necessary to carry out a survey to document the plants used for medicinal purposes in Babungo.

## Methods

### Study site

**Figure 1:**
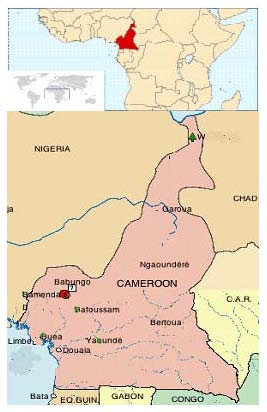
**Location of Babungo in Cameroon **[29].

The study was carried out in Babungo, located in the Ngoketunjia division of the Northwest Region of the Republic of Cameroon. Babungo falls between latitude 6° 01' and 6° 11' N and between longitudes 10° 20' and 10° 29' E [12] as shown in figure 1. The soil type is sandy clay ferruginous soil and the average altitude is 1200 m above sea level [13]. There are two seasons; the dry season from November to March and the rainy season from April to October. Annual precipitation is 2300 mm. Situated in the Sudan savannah, it is mostly covered with *Helictotrichon maitlandii *C.E Hubbard, *Andropogon gayanus *Kunth and *Pennisetum purpureum *[13]. Common tree species include *Cola *spp., *Spathodea campanulata *P. Beauv., *Markhamia lutea *(Benth) K. Schum., *Canarium schweinfurthii *Engl. and *Elaeis guinensis *Jacq [12]. The population, of about 14,000 inhabitants, are mostly peasant farmers who grow corn for subsistence and rice and coffee as cash crops. Blacksmithing and wood carving are also common occupations.

### Data collection

Traditional Medical Practitioners (TMP's) were the main informants in the survey. They were identified with the help of the traditional ruler and some of the elders of the tribe. A total of 17 TMP's were interviewed amongst whom 7 were literate. Their ages range from 28 to 70 years with more of them in the older side of the range. Most of the informants were men with only 4 females. After seeking their consent, the traditional medical practitioners were interviewed using semi-structure questionnaires and open-ended conversations. Trips were made to the sites where TMP's normally go to harvest plants and during such trips, there were discussions with the TMP's in addition to the interviews using the semi-structure questionnaires. The interviews and discussions were carried out in the local language since the author is a native speaker of the language. Data on the local names of the plants, the plant parts used, diseases treated by the plants, mode of usage and administration were collected in the field. Health conditions which were not recognized by the author were identified by the health professional at the health centre in Babungo. Plants recorded in the results were mentioned by at least two TMP's as treating the same disease in order to confirm its use. Fertile specimens of the plants were collected in the field using standard botanic methods [14] together with the TMP's. The collected specimens were identified at the National Herbarium in Yaoundé, Cameroon and voucher specimens were deposited at the herbarium of the Limbe Botanic Garden, Cameroon.

## Results

A total of 107 medicinal plant species from 98 genera and 54 families used for treating about 55 health problems were identified in the survey (see additional file 1). The most represented plant family in the list of medicinal plants is the Asteraceae with 12.1% of the medicinal plants species followed by Lamiaceae and Poaceae with 5.6% each.

Herbs made up 57% of the total number of medicinal plants followed by trees (24%), shrubs (15%), and climbers making up the remaining 4%. The leaves were the most commonly used plant part followed by the aerial parts of herbs (8%) and then by the bark (7%) of woody plants. The roots (6%), the flowers (5%), the fruits (4%) and latex (4%) are also used medicinally while the whole plant is the least used (3%).

Many plants (51.4%) have multiple medicinal uses and many diseases are treated using a combination of more than one plant (Additional file 1). For example venereal diseases are treated by oral administration of a concoction of the roots of *Capsicum frustescens, Vernonia amygdalina, C. schweinfurthii *and the bracts of *Musa paradisciaca*. For some species, the same part is used to treat different diseases. For example the leaves of *Carica papaya *are used for treating malaria and gastritis.

*C. papaya*, *Eucalyptus *spp., *Mangifera indica *and *Psidium guajava *do not have names in the local language and so their English names are used. Some plants from the Asteraceae family (*Ageratum conyzoides, Crassocephalum rubens, Dichrocephalla integrifolia, Erygerum floribundus and Vernonia calvoana*) are commonly called *ndobovensi *which translates to the devil's cigarette.

Decoctions were the most common method of preparation and most of the medicines are administered orally with topical administration in the case of skin diseases or wounds. The most commonly known and used plants are *Aspilia africana *to treat wounds, *Celosia globosa *to treat athlete's feet, *Cymbopogon citratus *for fevers and *Ocimum gratissimum *for stomachache.

Medicinal plants were harvested from the wild, from farms and fallows and some home gardens. From the list of medicinal plants (Additional file 1), 8.4% are cultivated exclusively for medicinal purposes (table 1), 19.6% are domesticated crops and fruits from farms, fallows or grown in compounds (table 2) and 72% are collected entirely from the wild. Forty five percent of medicinal plants (Additional file 1) have other non-medicinal uses as shown in table 3. For example wood from *Bersama abyssinica, C. schweinfurthii, Cordia platythyrsa*, *Croton macrostachus *are used for building houses, tool handles and furniture while *Annanas comosus, C. papaya, Citrus aurantium, M. indica *and *P. guajava *are common edible fruits.

**Table 1:** Plants cultivated solely for their medicinal uses.

Scientific name	Family
*Aloe vera *(L.) Burm.f.	Liliaceae

*Asystasia gangetica *(L.) T. Anders.	Acanthaceae

*Basella alba *L.	Basellaceae

*Cymbopogon citrates* (DC.) Stapf.	Poaceae

*Eremomastax speciosa* (Hochst.) Cufod.	Acanthaceae

*Ocimum gratissimum *L.	Lamiaceae

*Prunus africana *(Hook.f.) Kalkman	Rosaceae

*Senna alata *(L.) Roxb.	Caesalpiniaceae

*Voacanga africana *Stapf.	Apocynaceae

**Table 2:** A list of domesticated crops and fruits used for medicinal purposes.

Scientific name	Family
*Aframomum melegueta* K. Schum	Zingiberaceae

*Allium sativum *L.	Alliaceae

*Amaranthus hybridus *L.	Amaranthaceae

*Annanas comosus *(L.) Merr.	Bromeliaceae

*Capsicum annuum *L.	Solanaceae

*Carica papaya *L.	Caricaceae

*Citrus aurantium *L.	Rutaceae

*Citrus limon *(L.) Burm.f.	Rutaceae

*Cola acuminata *(P. Beauv.) Schott & Endl.	Sterculiaceae

*Elaeis guinensis *Jacq.	Arecaceae

*Mangifera indica *L.	Anacardiaceae

*Musa paradisiaca *L.	Musaceae

*Musa sapientum *L.	Musaceae

*Nicotiana tabacum *L.	Solanaceae

*Ocimum basilicum *L.	Lamiaceae

*Psidium guajava *L.	Myrtaceae

*Raphia hookeri *Man & Wendl.	Arecaceae

*Ricinus communis L*.	Euphorbiaceae

*Sorghum bicolor *(L.) Moench.	Poaceae

*Vernonia amygdalina* Delile	Asteraceae

*Zea mays *L.	Poaceae

**Table 3:** Non medicinal uses of medicinal plants in Babungo.

Scientific name	Family	Local name	Use
*Aframomum melegueta *K. Schum.	Zingiberaceae	Iswo	Used for driving away evil spirits.

*Afrostyrax kamerunensis *Perkins & Gilg	Huaceae	Fulong	Used as a spice.

*Agave sisalana *Perrine	Agavaceae	Nseng	Production of fibres.

*Allium sativum *L.	Alliaceae	Garlic	Used as a spice.

*Amaranthus hybridus *L.	Amaranthaceae	Fih	Eaten as a leaf vegetable.

*Annanas comosus *(L.) Merr.	Bromeliaceae	Pineapple	The fruits are edible.

*Bersama abyssinica *Fresen.	Melianthaceae	Fuaveti	Timber used for construction.

*Bidens pilosa *L.	Asteraceae	Shoctesuc	Whole plant is burn to repel insects.

*Bryophylum pinnatum* (Lam.) Oken	Crassulaceae	Juteweh	The juice from the leaves is used to remove stains.

*Caladium *spp.	Araceae	Lala	Ornamental plant.

*Canarium schweinfurthii* Engl.	Burseraceae	Tibew	The latex is burnt to drive away evil spirits. The fruits are edible and the wood is used as timber.

*Capsicum annuum *L.	Solanaceae	Nyanta	The fruits are added to food.

*Capsicum frutescens *L.	Solanaceae	Nyantafesucse	The fruits are added to food.

*Carica papaya *L.	Caricaceae	Pawpaw	The fruits are edible.

*Citrus aurantium *L.	Rutaceae	Orange	The fruits are edible.

*Citrus limon *(L.) Burm.f.	Rutaceae	Lemon	The fruit juice is added to some herbal teas.

*Cola acuminata *(P. Beauv.) Schott & Endl.	Sterculiaceae	Ibi	The seeds are served to guest to chew.

*Colocasia esculenta *(L.) Schott.	Araceae	Ndai	The tubers are edible.

*Cordia platythyrsa* Barker	Boranginaceae	Ibokwing	The wood is used for carving.

*Croton macrostachus* Hochst ex Delile	Euphorbiacea	Njang	The wood is used for carving, fuel wood and for tool handles.

*Cymbopogon citratus *(D.C) Stapf.	Poaceae	Ghaishek	The leaves are used for tea.

*Elaeis guinensis *Jacq.	Arecaceae	Iteh	The fruits used for making palm oil and the kernels for palm kernel oil. Palm wine is tapped from the tree.

*Entada abyssinica *Steud. ex A. Rich.	Mimosaceae	Fundung	The leaves are used for fodder.

*Eryngium foetidium *(L.) Urb.	Apiaceae	Bulung	The plant repels snakes.

*Eucalyptus *spp.	Myrtaceae	'Forest guide'	The wood is used for construction, furniture and for making xylophones.

*Ficus exasperata *Vahl	Moraceae	Ngwase	The leaves are used to scrub and clean kitchen utensils.

*Ficus thonningii *Blume	Moraceae	Ngung	Used for life fences.

*Jateorhiza macrantha* (Hook.f.) Exell & Mendonça	Menispermaceae		The leaves are used as toilet tissue in the bush.

*Kigelia africana *(Lam.) Benth.	Bignoniaceae	Thai	The wood is used for construction and tool handles.

*Lantana camara *L.	Verbenaceae	'Flower'	Ornamental plant.

*Mangifera indica *L.	Anacardiaceae	'Mango'	The fruit pulp is eaten.

*Markhamia lutea *(Benth.) K.Schum ex Engl.	Bignoniaceae	Bengtifua/ Tibeng	Provides shade. The wood is used for tool handles and carving.

*Musa paradisiaca *L.	Musaceae	Yuck	The fruits are eaten.

*Musa sapientum *L.	Musaceae	Nkwili	The ripe fruits are eaten.

*Nicotiana tabacum *L.	Solanaceae	Ndobo	The dried leaves are smoked.

*Ocimum basilicum *L.	Lamiaceae	Zwitefua	A decoction of the leaves is used to bath children who cry at night.

*Ocimum gratissimum *L.	Lamiaceae	Fulungfu	The plant serves as insect repellent.

*Piliostigma thonningii* (Schum.) Milne-Redh.	Caesalpiniaceae	Bing	The wood is used for construction.

*Polyscias fulva *(Hiern.) Harms	Araliaceae	Vai	The wood is used for making xylophones.

*Prunus africana *(Hook.f.) Kalkman	Rosaceae	'Kanda stick'	The wood is used for construction and for tool handles.

*Psidium guajava *L.	Myrtaceae	Guava	The fruit is edible.

*Raphia hookeri *G.Mann & H.Wendl.	Arecaceae	Kho	Sap from the palm is drunk as wine.

*Ricinus communis *L.	Euphorbiaceae	Medjai	The seeds are used in the production of castor oil.

*Sorghum bicolor *(L.) Moench	Poaceae	Saigini	The grains are edible.

*Spathodea campanulata* P.Beauv.	Bignoniaceae	Tibaibai	The wood is used for firewood and for making drums.

*Trema orientalis *(L.) Blume	Ulmaceae	Fai	The wood is used for building houses.

*Vernonia amygdalina *Delile	Asteraceae	Ying	The leaves are edible.

*Voacanga Africana *Stapf.	Apocynaceae	Thau	The seeds have a commercial Value.

*Zea mays *L.	Poaceae	Sai	The grains are the main staple food.

*Prunus africana *was reported by the TMP's to be extinct from the wild. A few stands of dead *P. africana *trees were seen during the field survey. Existing trees are found in private gardens and in some compounds.

Most of the TMP's were above 45 years old with only one less than 30 years old. Thus knowledge of the use of plants to treat diseases remains mostly with the older generation.

## Discussion

More plants from the family Asteraceae are used for medicinal purposes compared to any other plant family in Babungo because they contain a wide range of biologically active compounds and also because being one of the largest families in the plant kingdom, a large number of plants belong to this family [15,16]. The popularity of herbs in traditional medicine has been linked to their higher likelihood of containing pharmacologically active compounds compared to woody plant forms [16]. This may explain why more than half the plants recorded in the survey are herbs. Babungo is located in the grassland savannah which favours the growth of herbs. Most societies and cultures have a sound knowledge of the biodiversity in their environments as a result of long term experimentation and innovation [17]. This may explain the use of many herbs in the traditional medicinal practice in Babungo. Leaves of plants have been reported to accumulate, inulins, tannins and other alkaloids [18] which may be responsible for their medicinal properties, explaining its wide use. Other studies reported the leaves as the most widely used plant parts [[10,11], and [19]].

*Aloe vera *is used to treat malaria, gastritis, stomach ache, wounds and skin diseases, *Spilanthes filicaulis *is used to treat toothache, stomach ache, gastritis and malaria, *Lactuca capensis *is used to treat malaria, hypertension and gastritis. This may be because some plants contain many secondary metabolites which could have different pharmacological activities and consequently treat different diseases. The TMP's believe that combining more than one plant re-enforces the medicines, increasing their effectiveness. Similar findings have been reported in Ethiopia [20]. Pharmacological studies supported this believe [21]. However the same study reported that combining some drugs could have antagonistic effects.

*A. africana *has been found to contain phytochemicals which are capable of arresting wound bleeding, preventing the growth of wound contaminating microbes and accelerating wound healing [22]. Similar phytochemical analyses have been made for *C. citratus *[23] and *O. gratissimum *[24] and they were found to contain chemicals which relieve fevers and stomach ache respectively. This scientifically validates their common use for these purposes in Babungo.

The fact that many medicinal plants have other uses may lead to their over exploitation, threatening their continuous survival in the area. Not many medicinal plants were cultivated solely for their medicinal values. This is because most people will prefer to cultivate food or cash crops rather than medicinal plants since most medicinal plants are either not sold or sell at very low prices and therefore not profitable, providing very little incentives for their cultivation.

Some exotic species do not have names in the local language and were called by their English names. This is because these are recently introduced species in the area. In some cases, the names of plants in the local language were descriptive of some character of the plant; *S. filicaulis *called *nyantanyui *literally translating to God's pepper because of its pepper-like tasting flowers. The grouping of the some Asteraceae under one common name could reflect the local system of plant classification.

The use of the bark of *P. africana *in the local traditional medicine and exploitation for commercial purposes has resulted to the extinction of the species from the wild. The bark was harvested and sold to Plantecam, a company which exported the bark or its processed extracts to Europe for manufacture of drugs used to treat benign prostatic hyperplasia sold under the brand name "Tadenan" (France) or "Pygenil" (Italy) [25]. *P africana *has been listed by the International Union for the Conservation of Nature (IUCN) on its redlist of threatened species [26] for which conservation action must be taken.

Many of the medicinal plants used in Babungo have been reported in other areas with similar or different uses; 12 species were found to be used in the Mount Cameroon area [9], 17 species are used in the neighbouring Fundong subdivion [11] and 19 species in Aguambu [10]. One species, *Vernonia calvoana*, has been cited as a new entrant in the list of medicinal plants in Cameroon [11]. Four species were listed in Kenya [27] while 3 and 13 species were documented in the mid-west and south-east of Ethiopia respectively [20,28]. The use of medicinal plants across cultures and wider geographic regions has been discussed as prove validating their medicinal properties [28]. The chemical composition of some of the plants reported in this study has been scientifically studied. The root of *Rauvolfia vomitoria*, used traditionally for high blood pressure, has been found to contain reserpine which lowers the blood pressure and slows down the heartbeat while the flowers of *S. filicaulis *were found to contain spilanthol a local anaesthetics [9]. The seeds if *C. acuminate *has been reported to contain 2.5% caffeine, which is known to stimulate the central nervous system [5]. This explains why cola nuts are chewed as a stimulant by the people of Babungo.

Most young people are not interested in traditional medical practice because it is less profitable compared to growing cash crops. The influence of western culture, rural-urban migration in search for better educational and job opportunities and the commonly held view by young people that traditional medicine is superstitious and something for the poor and uneducated may result to a loss of this rich and useful knowledge which has accumulated over several generations.

## Conclusions

The survey shows that a large number of medicinal plants are used in Babungo for treating different ailments. The knowledge of the use of plants to treat diseases has been with the people for generations but has not been recorded. This knowledge remains mostly with the traditional medical practitioners who are mostly old people. Most of the medicinal plants are sourced from the wild. In addition to their medicinal uses, some of these plants have other uses. The local population should be educated on sustainable methods of harvesting plants to treat diseases today without compromising their availability for future use. The youth should also be encouraged to learn the traditional medicinal knowledge to preserve it from being lost with the older generation.

## Competing interests

The author declares that they have no competing interests.

## Authors' contributions

Being the sole author, DJS initiated the idea, developed the questionnaire, carried out the survey and wrote the article.

## Supplementary Material

Additional file 1 **Medicinal plants used in Babungo for treating different diseases.** The additional file list botanical and local names of the plants, the plant part used, the use and the preparation and mode of administration. Click here for file

## References

[B1] Balick MJ Cox PA Plants, People, and Culture: The Science of Ethnobotany 1996 Scientific American Library: New York

[B2] Rates SMK Plants as a source of drugs Toxicon 2001 39 603 613 10.1016/S0041-0101(00)00154-9 11072038

[B3] WHO Traditional medicine Fact sheet No 134 2003

[B4] Muthu C Ayyanar M Raja N Ignacimuthu S Medicinal plants used be traditional healers in Kancheepuram District of Tamil Nadu, India J Ethnobio Ethnomed 2006 2 43 10.1186/1746-4269-2-43 PMC1615867 17026769

[B5] Hostettmann K Marston A Ndjoko K Wolfender J-L The Potential of African Medicinal Plants as a Source of Drugs Current Organic Chemistry 2000 4 973 1010 10.2174/1385272003375923

[B6] Sofowora A Medicinal plants and traditional medicine in Africa 1993 Spectrum books limited: Ibadan8510470

[B7] Vulto AG Smet PAGM Dukes MMG Drugs used in non-orthodox medicine Meyler's side effects of drugs 1988 11 Elsevier: Amsterdam 999 1005

[B8] Thomas DW Thomas JM Bromley WA Mbenkum FT Korup Ethnobotany survey Final report to the World Wide Fund for Nature 1989 Surrey: UK

[B9] Ndenecho EN Herbalism and resources for the development of ethnopharmacology in Mount Cameroon region Afr J Pharm Pharmacol 2009 3 3 078 086

[B10] Focho DA Ndam WT Fonge BA Medicinal plants of Aguambu-Bamumbu in the Lebialem highlands, southwest province of Cameroon Afr J Phrm Pharmacol 2009 3 1 1 13

[B11] Focho DA Newuh MC Anjah MG Nwana FA Ambo FB Ethnobotanical survey of trees in Fundong, Northwest Region, Cameroon J Ethnobiol Ethnomed 2009 5 17 10.1186/1746-4269-5-17 19555468 PMC2708145

[B12] MINEF Annual report of activities carried out by the Divisional Section for Forestry, Ngokentunjia 1999 Ministry of Environment and Forest

[B13] Ndenecho EN Cropping Systems and Post-Cultivation Vegetation Successions: Agro-Ecosystems in Ndop, Cameroon J Hum Ecol 2009 27 1 27 33

[B14] Olorode O Taxonomy of West African Flowering Plants 1984 Longman: London

[B15] Heinrich M Robles M West JE Ortiz de Montellano BR Rodriguez E Ethnopharmacology of Mexican Asteraceae (Compositae) Annual Review of Pharmacology and Toxicology 1998 38 539 565 10.1146/annurev.pharmtox.38.1.539 9597165

[B16] Thomas E Vandebroek I Sanca S Van Damme P Cultural significance of medicinal plant families and species among the Quechua farmers in apillampampa, Bolivia Journal of Ethnopharmacology 2009 122 60 67 10.1016/j.jep.2008.11.021 19101618

[B17] Dovie BK Witkowski ET Shackleton CM Knowledge of Plant Resource use Based on Location, Gender and Generation Applied Geography 2008 28 311 322 10.1016/j.apgeog.2008.07.002

[B18] Okoegwale EE Omefezi JU Some herbal preparations among the people of Isoko Clan of Delta State, Nigeria J Appl Sc 2001 4 2350 2371

[B19] Signorini MA Piredda M Bruschi P Plants and traditional knowledge: An ethnobotanical investigation on Monte Ortobene (Nuoro, Sardinia) J Ethnobiol Ethnomed 2009 5 6 10.1186/1746-4269-5-6 19208227 PMC2661884

[B20] Flatie T Gedif T Asres K Gebre-Mariam T Ethnomedical survey of Berta ethnic group Assosa Zone, Benishangul-Gumuz regional state, mid-west Ethiopia J Ethnobiol Ethnomed 2009 5 14 10.1186/1746-4269-5-14 19409096 PMC2688485

[B21] Chow KU Nowak D Boehrer S Ruthardt M Knau A Hoelzer D Mitrou PS Weidmann E Synergistic effects of chemotherapeutic drugs in lymphoma cells are associated with down-regulation of inhibitor of apoptosis proteins (IAPs), prostate-apoptosis-response-gene 4 (Par-4), death-associated protein (Daxx) and with enforced caspase activation Biochemical Pharmacology 2003 66 711 724 10.1016/S0006-2952(03)00410-6 12948851

[B22] Okoli CO Akah PA Okoli AS Potentials of leaves of *Aspilia africana *(Compositae) in wound care: an experimental evaluation BMC Complementary and Alternative Medicine 2007 7 24 10.1186/1472-6882-7-24 17623087 PMC1940021

[B23] Olaniyi AA Sofowora EA Oguntimehin BO Phytochemical investigation of some Nigerian plants used against fevers. II. *Cymbopogon citratus* Planta Med 1975 28 2 186 189 10.1055/s-0028-1097851 1197425

[B24] Akinyemi KO Oladapo O Okwara CE Ibe CC Fasure KA Screening of crude extracts of six medicinal plants used in South-West Nigerian unorthodox medicine for anti-methicillin resistant *Staphylococcus aureus *activity BMC Complementary and Alternative Medicine 2005 5 6 10.1186/1472-6882-5-6 15762997 PMC1079793

[B25] Cunningham AB Mbenkum FT Sustainability of harvesting of *Prunus Africana *bark: A Medicinal Plant in International Trade People and Plants working paper 2 1993 Paris: Unesco

[B26] IUCN 2009 IUCN Red list of Threatened Species Version 2009.2 Downloaded on 05 December 2009 http://www.iucnredlist.org

[B27] Bussmann R Ethnobotany of the Samburu of Mt. Nyiru, South Turkana, Kenya J Ethnobiol Ethnomed 2006 2 35 10.1186/1746-4269-2-35 16956401 PMC1570449

[B28] Lelukal E Kelbessa E Bekele T Yineger H An ethnobotanical study of medicinal plants in Mana Angetu District, southeastern Ethiopia J Ethnobiol Ethnomed 2008 4 10 10.1186/1746-4269-4-10 18442379 PMC2391147

[B29] Fonjong LN Mbah FA The Fortunes and Misfortunes of Women rice Producers in Ndop, Cameroon and the Implications for Gender Roles Journal of International Women Studies 2007 8 4 133 147

