# Supplementary material for: An ethnobotanical survey of medicinal plants in Babungo, Northwest Region, Cameroon
Source: J Ethnobiol Ethnomed. 2010 Feb 15;6:8. doi: 10.1186/1746-4269-6-8 (PMC2843657; doi:10.1186/1746-4269-6-8)
Supplement: Additional file 1 — Medicinal plants used in Babungo for treating different diseases. The additional file list botanical and local names of the plants, the plant part used, the use and the preparation and mode of administration. [file 1746-4269-6-8-S1.PDF]

Additional file 1: Medicinal plants used in Babungo for treating different diseases.

Botanical name	Family	Local name	Voucher specimen number	Part used	Use	Preparation and administration
Acanthus montanus (Nees) T. Anders.	Acanthaceae	Gekbou	DS 127	Leaves	Malaria	Concoction with the leaves of *Senna alata* is taken orally.
Aframomum melegueta K. Schum	Zingiberaceae	Iswo	DS 193	Seeds	Component of many herbal remedies	Used in combination with other herbs.
Afrostyrax kamerunensis Perk and Gilg.	Huaceae	Fulong	DS 135	Seeds	Venereal diseases	Concoction with the roots of *Raphia hookeri* is taken orally.
Agave sisalana Perrine	Agavaceae	Nseng	DS 187	Leaves	Local analgesic and anti-inflammatory	The paste from ground leaves is applied topically on affected parts.
Ageratum conyzoides L.	Asteraceae	Ndobovensi	DS 169	Stem, leaves and flowers	Bronchitis/ Fevers	An infusion of the aerial part of the plant is taken orally.
				Leaves	Infantile diarrhoea	Maceration of the young leaves is taken orally.
Aloe vera (L.) Burm.f.	Liliaceae	Aloe vera	DS 202	Leaves	Gastritis/ Stomachache	Leaves are chewed and the leaf juice is swallowed.
					Malaria	Maceration is taken orally.
				Latex	Wounds/ Skin diseases	Latex from the leaves is applied on affected parts.
Allium sativum L.	Alliaceae	Garlic	DS 175	Leaves	Hypertension	The bulbs are added to a decoction of the leaves of *Lantana camara* and taken orally.
Amaranthus hybridus L.	Amaranthaceae	Fih	DS 183	Leaves	Anti-inflammatory	Paste from ground leaves are applied on affected parts.
Amaranthus lividus L.	Amaranthaceae	Flower bai	DS 166	Leaves	Aneamia	Leaves are squeezed and the leaf juice taken orally.
Annanas comosus (L.) Merr.	Bromeliaceae	Pineapple	DS 170	Leaves	Malaria	Concoction of the leaves *A. comosus* and

						the stem bark of *Spathodea campanulata* is taken orally.
Aspilia africana (Pers) C. D. Ada	Asteraceae	Wowoh	DS 172	Leaves	Wounds	Leaf juice from crushed leaves is applied on wounds.
					Gastritis	Young leaves are chewed and the leaf juice is swallowed.
Asystasia gangetica (L.) T. Anders.	Acanthaceae	Formenshi	DS 148	Leaves	Aneamia	The leaves are squeezed and the leaf juice is taken orally.
Basella alba L.	Basellaceae	Luii	DS 180	Stem and leaves	Strengthen neonates	Decoction is taken as an enema.
Bersama abyssinica Fresen.	Melianthaceae	Fuaveti	DS 131	Stem bark	Stomachache	Decoction of the stem bark is taken orally.
Bidens pilosa L.	Asteraceae	Shoctesuc	DS 176	Whole plant	Malaria	Concoction with the leaves of *Cymbopogon citratus* is taken orally.
					Insecticide	Whole plant is burnt to keep away insects.
				Leaves	Wounds	Leaves are crushed and leaf juice applied on wounds.
Bryophylum pinnatum (Lam.) Oken	Crassulaceae	Juteweh	DS 225	Leaves	Painful ear	Leaves are warmed and the leaf juice is squeezed into the ears.
Caladium sp.	Araceae	Lala	DS 161	Leaves	Malaria	Decoction taken orally.
Canarium schweinfurthii Engl.	Burseraceae	Tibew	DS 226	Root	Venereal diseases/ Aphrodisiac	Concoction with the roots of *Vernonia amygdalina*, *Capsicum frustesens* and the bracts of *Musa paradisiaca* is taken orally.
Capsicum annuum L.	Solanaceae	Nyanta	DS 125	Fruit	Restorative after delivery	Fruits are added to soups in large quantities.
Capsicum frutescens L.	Solanaceae	Nyantafesucse	DS 186	Fruit	Gastritis	Added to a concoction to treat gastritis.

				Roots	Venereal disease/ aphrodisiac	Concoction with the bracts of *M. paradisiaca* and the roots of *C. schweinfurthii, V. amygdalina*, and *C. frutescens* is taken orally.
Carica papaya L.	Caricaceae	Pawpaw		Leaves	Malaria	Leaf juice is squeezed and taken orally.
					Gastritis	Leaf juice is mixed with grated *Allium sativum* bulbs and the fruit juice of *Citrus limon* and taken orally.
					Jaundice	Concoction with the yellowing leaves of *M. paradisiaca* is taken orally.
					Wounds	Leaf juice is applied on fresh wounds.
				Roots and leaves	Typhoid	Decoction of a mixture of the roots and leaves is taken orally.
Cassia hirsuta L.	Caesalpiniaceae	Tulushine		Leaves	Skin diseases	Decoction is taken orally.
Celosia globosa Schinz.	Amaranthaceae	Mumebeng	DS 182	Leaves	Athlete's foot	Leaves are squeezed and the leaf juice applied on affected parts.
					Navel pains	A paste made from a mixture of ground leaves and palm oil and taken orally.
Centella asiatica (L.) Urb.	Apiaceae	Nid	DS 218	Leaves	Diabetes	Infusion of the leaves is taken orally.
Chenopodium ambrosioides L.	Chenopodiaceae	Fulunfu	DS 209	Leaves	Convulsion	Leaf juice administered orally.
Chromolaena odorata (L.) R.M. King and H. Robinson	Asteraceae	Twigi	DS 149	Leaves	Wound dressing/skin diseases	Leaf juice is applied on wounds and on skin diseases. A poultice made from the crushed leaves is used to cover wounds.

Citrus aurantium L.	Rutaceae	Orange	DS 143	Roots and leaves	Malaria/jaundice	Decoction of the roots and leaves is taken orally.
Citrus limon (L.) Burm.f.	Rutaceae	Lemon	DS 147	Fruit	Gastritis	The fruit juice is added to *C. papaya* leaf juice and taken orally.
Clematopsis scabiosifolia (D.C) Hutch.	Ranunculaceae	Nyantatoh	DS 141	Stem and leaves	Stimulant	Infusion taken orally.
Cola acuminata (P. Beauv.) Schott & Endl.	Sterculiaceae	Ibi	DS 221	Seeds	Stimulant	The seeds are chewed.
				Leaves	Diarrhoea	Leaves are squeezed and leaf juice taken orally.
Cola nitida (Vent.) Schott & Endl.	Sterculiaceae	Ibi	DS 184	Same as above	Same as above	Same as above
Colocasia esculenta (L.) Schott.	Araceae	Ndai	DS 161	Tubers	Whitlow	Paste from grated tubers is applied on affected parts and tied with a band.
Commelina benghalensis L.	Commelinaceae	Wiwih	DS 227	Latex	Ring worm	Latex is applied on affected parts.
				Leaves	Headache	Poultice made from the warm leaves is pressed and rubbed on the forehead.
Cordia platythyrsa Barker	Boranginaceae	Ibokwing		Young leaves	Cough and tuberculosis	Decoction is administered orally.
Crassocephalum rubens (Juss. Ex.Jacq.) S. Moore.	Asteraceae	Ndobovensi	DS 178	Leaves	Conjunctivitis	Juice from the crushed leaves is dropped into the infected eye.
Crotalaria mucronata Desv.	Papilionaceae	Nekbaba	DS 192	Leaves	Convulsion	Paste from ground leaves is mixed with palm oil and taken orally.
Croton macrostachyus Hochst. ex Del.	Euphorbiaceae	Njang	DS 133	Root	Purgative	Decoction is administered orally.
Cymbopogin citratus (DC.) Stapf.	Poaceae	Fever grass	DS 216	Leaves	Fever/Malaria	Concoction of the leaves with the leaves of *B. pilosa* is taken orally.
Dichrocephalla integrifolia	Asteraceae	Ndobevensi	DS 181	Stem and leaves	Headache	Warmed leaves are pressed on the

(L.f.) Kuntze						forehead.
Elaeis guinensis Jacq.	Arecaceae	Iteh	DS 190	Fruits	Poison antidote	Palm oil made from the fruit is taken orally.
Eleusine indica (L.) Gaertn.	Poaceae	Woh	DS 207	Leaves	Side pains	Pounded leaves are mixed with palm oil and taken orally.
					Fractures	Paste from pounded leaves is applied on broken parts and tied with a bandage.
Emilia coccinea (Sims.) G. Don	Asteraceae	Nsefouse Femefous e	DS 152	Leaves	Gastritis	Leaves are chewed and leaf juice swallowed.
					Wounds	Leaves are crushed and leaf juice is applied on wounds.
					Suppuration of the ears	Leaves are warmed and leaf juice is squeezed into the ears.
Ensete gilletii (De Wild.) Cheesman	Musaceae	Ngomeke	DS 126	Roots	Mental illness	Decoction is taken orally.
Entada abyssinica Steud. Ex. A. Rich.	Mimosaceae	Fundung	DS 146	Stembark	Malaria	Decoction is taken orally.
Eremomastax speciosa (Hochst.) Cufod.	Acanthacea e	Nsebai	DS 223	Leaves	Aneamia/Sto machache	Leaves are squeezed and leaf juice taken orally.
Erygeron floribundus (H.B. and K.) Sch.Bip	Asteraceae	Ndoboven si	DS 156	Leaves	Gastritis/mens trual pains	Young leaves are chewed and the leaf juice is swallowed.
Eryngium foetidium L.	Apiaceae		DS 184	Leaves	Irregular heart beat	Leaves are squeezed and the juice taken orally.
Erythrina senegalensis DC.	Papilionace ae	Tinyao	DS 196	Stem bark	Jaundice	Decoction of the stem bark is taken orally.
Eucalyptus spp.	Myrtaceae	Forest guide	DS 128	Young leaves	Fevers and colds	Infusion of the young leaves is taken orally.
Euphorbia hirta L.	Euphorbiace ae	Femenven si	DS 132	Stem, leaves and flowers	Gastritis Stomachache Nausea	Stem, leaves are flowers are chewed and juice swallowed.
Ficus exasperata Vahl.	Moraceae	Ngwase	DS 204	Fruit	Female infertility	Decoction of the fruit is taken orally.
Ficus thonningii	Moraceae	Ngung	DS DS	Stem bark	Malaria	Concoction with the

Blume			127			leaves of *C. citratus* is taken orally.
Gossypium hirsutum L.	Malvaceae	Meh		Leaves	Venereal diseases	A decoction of the leaves is taken orally.
				Roots	Malaria	Decoction of the roots is taken orally.
Harungana madagascariensis Lam. ex Poir.	Hypericaceae	Febai		Leaves	Filaria	Leaves are crushed and leaf juice applied on affected parts.
Hemizygia welwitschii (Rolfe.) M. Ashby	Lamiaceae	Yengong		Stem, leaves and flowers	Malaria	Decoction of the stem, leaves and flowers is taken orally.
Hibiscus cannabinus L.	Malvaceae	Viguala		Leaves	Aneamia	Leaves are squeezed and the leaf juice taken orally.
Hilleria latifolia (Lam) H. Walt.	Phytolaccaceae	Bibe		Stem, leaves and flowers	Strengthen neonates	Decoction is used as enema and also for a bath.
Hydrocotyle bonariensis Lam.	Apiaceae	Itomau		Whole plant	Navel pains	Decoction of the whole plant is taken orally.
Jateorhiza macrantha (Hook.f.) Exell & Mendoca	Menispermaceae	Gaiko		Leaves	Venereal disease	Concoction of the bark with the seeds of *A. melegueta* is taken orally.
Jetropha curcas L.	Euphorbiaceae	Medjai		Roots	Venereal diseases	Decoction of the roots is taken orally.
				Latex	Wounds	Latex from cut stem applied on wounds.
					Skin diseases	Latex from cut stem applied on affected parts.
Kigelia africana (Lam.) Benth.	Bignoniaceae	Thai		Stem bark and leaves	Rheumatism	Decoction of the stem and leaves is taken orally.
Lactuca capensis Thunb.	Asteraceae	Nsefouse/ Femeh		Leaves	Cerebral malaria	Decoction of the leaves is taken orally.
					Gastritis	Leaves are chewed and the leaf juice is swallowed.
					Hypertension	Decoction of the leaves

						is taken orally.
Lannea schimperi (Hochst. ex. A.Rich.) Engl.	Anacardiaceae	Nkwelegito		Stem bark	Toothache	Decoction of the stem bark is used as a mouth wash
Lantana camara L.	Verbenaceae	Flower		Leaves	Typhoid	Decoction of the leaves is taken orally.
					Hypertension	*A. sativum* bulbs are added to a decoction of the leaves and taken orally.
Leonotis nepetifolia (L.) R.Br.	Lamiaceae	Fulong		Leaves	Side pains	Decoction of the leaves is taken orally.
Mangifera indica L.	Anacardiaceae	Mango		Stem bark and leaves	malaria	Concoction of the stem bark and the leaves of *C. citratus* and *Psidium guajava* is taken orally.
Markhamia lutea (Benth.) K.Schum. ex Engl.	Bignoniaceae	Bengtifua/ Tibeng		Stem bark and leaves	Rheumatism	Decoction of the stem and leaves is taken orally.
Mikania cordata (Burm.f.) B.L. Robinson	Asteraceae	Shobise		Leaves	Stomachache	Leaves are squeezed and leaf juice taken orally.
					Abortifacient	Leaves are squeezed and leaf juice taken orally.
Mimosa invisa L.	Mimosaceae	Kwiteweh		Leaves	Whitlow	Crushed leaves are applied on affected parts.
Mimosa pudica L.	Mimosaceae	Kwiteweh		Leaves	Palpitation	Leaves are chewed with palm kernels.
Mormodica cissoides Planche. ex. Benth.	Cucurbitaceae	Woteh		Leaves	Navel pains	Decoction of the leaves is used for enema.
					Constipation	Decoction of the leaves is used for enema.
Musa paradisiaca L.	Musaceae	Nkwili		Bracts	Aphrodisiac/ Venereal disease	Concoction of the bracts, the roots of *C. Schweinfurthii*, *V. amygdalina* and *C. frutescens* is taken orally.
				Leaves	Jaundice	Concoction with the yellowing leaves of *C. papaya* taken orally.

Musa sapientum L.	Musaceae	Yuck		Bracts	Filaria	Decoction of the bracts is taken orally.
				Fruit	Cough	The rind is burnt and the resulting ash is mixed with palm oil and administered orally.
Nicotiana tabacum L.	Solanaceae	Ndobo		Leaves	Toothache	Dried leaves are ground into a fine powder and put on painful tooth.
					Stimulant	Dried leaves are smoked.
Ocimum basilicum L.	Lamiaceae	Zwitefua		Leaves	Constipation	Infusion of the leaf is taken orally.
Ocimum gratissimum L.	Lamiaceae	Fulungfu		Leaves	Stomachache	Leaves are squeezed and the leaf juice taken orally.
					Convulsion in children	Leaves are squeezed and the leaf juice are dropped into the eyes and rubbed all over the body.
Oxalis corniculata L.	Oxalidaceae	Ithomau		Stem and leaves	Navel pains	Decoction of the stem and leaves is taken orally.
Paspalum conjugatum Berg.	Poaceae	Shocsi		Whole plant	Venereal diseases	Concoction of the whole plant, *Stelleria media*,and *Spilanthes filicaulis* is taken orally.
Physalis angulata L.	Solanaceae	Batetoh		Fruits	Restorative after delivery	Ripe fruits are eaten fresh
Piliostigma thonningii (Schum.) Milne-Redhead.	Caesalpiniaceae	Bing		Stem bark	Malaria	Decoction of the stem bark is taken orally.
Piper umbellatum L.	Piperaceae	Mumbua		Leaves	Piles	Leaf juice is rubbed around the anus.
Plactranthus glandulosus Hook.f.	Lamiaceae	Nsebai		Leaves	Aneamia	Leaves are squeezed and the leaf juice is taken orally.
					Diarrhoea	Leaves are squeezed and the leaf juice is taken orally.
Platycerium stemaria (P. Beauv.) Desv.	Polypodiaceae	Isocnekai		Leaves	Stomachache	Leaves are dried in a hot pot, ground into a

						fine powder, mixed with palm oil and taken orally.
Polyscias fulva (Hiern.) Harms	Araliaceae	Vai		Leaves	Rheumatism	Decoction of the leaves is taken orally.
Polystachya odorata Lindl.	Orchidaceae			Whole plant	Stomachache	Whole plant is dried in a hot pot, ground into a fine powder, mixed with palm oil and taken orally.
Portulaca oleracea L.	Portulacaceae	Ngekbaba		Stem and leaves	Venereal diseases	Concoction with the seeds of *A. melegueta* is taken orally.
Prunus africana (Hook.f.) Kalkman	Rosaceae	Pygeum		Stem bark	Mental illness	Decoction of the stem bark is taken orally.
					Fever	Decoction of the stem bark is taken orally.
Psidium guajava L.	Myrtaceae	Guava		Leaves	Diarrhoea	Young leaves are chewed and the leaf juice swallowed.
					Malaria	Concoction with the leaves of *M. indica*, the leaves of *C. citratus* is taken orally
Raphia hookeri Man & Wendl.	Arecaceae	Kho		Roots	Venereal disease	Concoction with the fruits of *A. kamerunensis* is orally.
				Sap	Induces lactation	Fermented sap from the tree is drunk.
Rauvilfia vomitoria Afzel.	Apocynaceae	Thauvengo		Roots	High blood pressure/ Mental illness	Decoction of the roots is taken orally.
Ricinus communis L.	Euphorbiaceae	Medjai		Seeds	Vermifuge	Castor oil extracted from the seeds is administered orally to expel intestinal worms.
Sanseviera liberica Ger. & Labr.	Agavaceae	Nseng	DS 164	Leaves	Anti-inflammation	Leaves are ground into a paste which is applied on affected parts.
Scoparia dulcis L.	Scrophulariaceae	Fornyui		Leaves	Poison antidote	Leaves are chewed and the leaf juice is swallowed.
					Traditional elixir	Dried in a hot pot and ground into a powder.
Senna alata (L.)	Caesalpiniace	Forgwinan		Leaves	Malaria	Concoction of the

Roxb.	ae	ase				leaves with those of *A. montanus* is administered orally.
Sida rhombifolia L.	Malvaceae	Nsengnebew		Young leaves	Drowsiness	Leaves are chewed and the leaf juice is swallowed.
Sida veronicifolia L.	Malvaceae	Mbuase		Leaves	Eases delivery	Leaves are squeezed and the leaf juice taken orally.
Sorghum bicolor (L.) Moench.	Poaceae	Saigini		Stem and leaves	Filaria	Decoction of the stem and leaves is taken orally.
Spathodea campanulata P. Beauv.	Bignoniaceae	Siale/Tibaibai		Stem bark	Malaria/ Venereal diseases	Decoction of the stem bark is taken orally.
Spilanthes filicaulis (Schum. and Thonn.) C.D. Ada.	Asteraceae	Nyantanyui		Flower	Toothache	A flower is chewed using the painful tooth.
				Stem, leaves and flowers	Stomachache/ Gastritis	Stem, leaves and flowers are chewed and the leaf juice is swallowed
					Malaria	Stem, leaves and flowers are a component of a mixture of plants used to treat malaria.
Stellaria media (L.) Vill.	Caryophyllaceae	Fotofoteyese		Leaves and stem	Coccidiosis	The leaves are crushed and the leaf juice is feed to birds.
					Venereal diseases	Concoction of the stem, leaves and flower combined with *S. filicaulis* and *P. conjugatum* is taken orally.
Thevetia neriifolia Juss.	Apocynaceae	Thau		Leaves	Local analgesic	Leaves are ground into a paste which is applied on affected parts.
Trema orientalis (L.) Blume	Ulmaceae	Fai		Leaves and stem bark	Irregular heart beat	Decoction taken orally.
Vernonia amygdalina Delile	Asteraceae	Ying		Leaves	Jaundice/malaria	Concoction with the leaves of *C. papaya* is taken orally.

					Wound dressing	Poultice made from the crushed leaves is used to cover wounds.
					Venereal diseases/aphrodisiac	Concoction with the roots of *C. schweinfurthii* and *C. frutescens* is taken orally.
Vernonia calvoana var *microcephala* C.D. Adams	Asteraceae	Ndobovensi		Leaves and stem	Headache	Leaves and stems are warmed over a fire and pressed on the forehead.
Voacanga africana Stapf.	Apocynaceae	Thau		Stem bark and roots	Mental illness	Concoction of the stem bark and the roots is taken orally.
Zea mays L.	Poaceae	Sai		Silk	Malaria	Infusion of the silk and the fruit juice of *C. limon* is taken orally
Zehneria scabra (L.f.) Sond.	Cucurbitaceae	Gi		Leaves	Cryptorchidism	Decoction of the leaves is used for enema.
